# Correlations between capability face pressure, job crafting, and burnout among orthopedic nurses

**DOI:** 10.3389/fpubh.2025.1727198

**Published:** 2026-01-15

**Authors:** Li Zhang, Hongbing Ba

**Affiliations:** 1 BAYI Orthopedic Hospital, China RongTong Medical Healthcare Group Co. Ltd, Chengdu, China; 2 School of Physical Education and Health, Aba Teachers College, Shuimo, China

**Keywords:** burnout, capability face pressure, job crafting, mediating effect, orthopedic nurse

## Abstract

**Objective:**

This study aims to determine the current status of capability face pressure (CFP; psychological stress derived from the gap between one’s ability and external expectations), job crafting, and burnout among orthopedic nurses; reveal their correlations and the mediating effect of job crafting; and provide empirical support for developing targeted burnout intervention strategies in this field.

**Methods:**

A total of 216 orthopedic nurses from Sichuan Province, China, were enrolled in this study. Participants completed the CFP Scale, the Job Crafting Scale, and the Maslach Burnout Inventory–Human Services Survey (MBI-HSS). Pearson’s correlation coefficients were calculated to examine the associations among variables. The bootstrap method was used to test the significance of the mediating effect.

**Results:**

The mean CFP score was 13.86 ± 2.62, and the mean job crafting score was 88.72 ± 14.95. The total burnout score was 58.64 ± 10.52. CFP differed significantly across sex, age, education, professional title, years of experience, income, and number of night shifts (all *p* < 0.05). Burnout was positively associated with CFP (*r* = 0.658, *p* < 0.01) and negatively associated with job crafting (*r* = −0.576, *p* < 0.01). Job crafting partially mediated the relationship between CFP and burnout, with an indirect effect accounting for 42.87% of the total effect.

**Conclusion:**

CFP is significantly positively correlated with burnout, whereas job crafting is significantly negatively correlated with burnout among orthopedic nurses. Job crafting exerts a partial mediating effect between CFP and burnout, with the indirect effects constituting 42.87% of the total effect. Reducing CFP through targeted psychological support and capability recognition, enhancing job crafting via specialized training and collaborative support, and focusing on nurses aged 30–40 years, those with intermediate professional titles, and those working frequent night shifts may alleviate burnout among orthopedic nurses.

## Introduction

Burnout is a psychological syndrome marked by emotional exhaustion, depersonalization, and a reduced sense of personal accomplishment, resulting from prolonged exposure to occupational stress (1, 2). Orthopedic nursing is a high-risk, high-demand specialty in clinical care. Patients are typically recovering from fractures or joint replacement surgeries and require extended bed rest. Recovery is often prolonged, with common complications including deep vein thrombosis and infection (3, 4). Orthopedic procedures such as scoliosis correction and joint revision are technically complex, requiring nurses to perform specialized tasks, including wound care and rehabilitation support. Additionally, the unpredictability of emergency trauma cases requires nurses to maintain a constant state of vigilance (5, 6). These factors, coupled with the fact that orthopedic nurses care for 8–10 postoperative patients daily (with repetitive high-intensity tasks such as wound care and rehabilitation guidance accounting for over 60% of their work) and generally work more than 10 h per day (5), place them at high risk of burnout. This can impair work efficiency, compromise care quality, and contribute to increased turnover and staffing shortages, making the alleviation of burnout among orthopedic nurses an urgent priority in nursing management.

Fracture patients have a long rehabilitation cycle (average 3–6 months), and nurses must continuously address the emotional needs, such as anxiety and rehabilitation expectations, of patients and their families, resulting in a significantly higher emotional labor load than general departments (6). Moreover, the nurse–patient ratio in orthopedic departments of Grade A tertiary hospitals in China is generally lower than 1:0.4 (7), and staffing shortages force nurses to often take on cross-post tasks, such as emergency trauma management and postoperative complication monitoring, further exacerbating burnout due to organizational pressure. These factors place orthopedic nurses at a high risk of burnout, which can impair work efficiency, compromise care quality, and contribute to increased turnover and staffing shortages. Therefore, alleviating burnout among orthopedic nurses has become an urgent priority in nursing management.

Capability face pressure (CFP) (8, 9) refers to the psychological stress experienced by individuals in occupational settings due to the perceived gap between their own ability and external expectations (e.g., professional identity, task requirements, and the need for others’ approval). Its core feature is the interaction between face concerns and ability cognition. Unlike self-efficacy (confidence in one’s own ability), which emphasizes confidence in internal capabilities, CFP focuses on stress derived from external expectations; unlike emotional labor (regulating emotions to meet work requirements), which centers on emotional regulation, CFP highlights face-threatening stress caused by ability gaps (8–10).

The high precision (e.g., rehabilitation guidance after joint replacement) and high responsibility (e.g., early warning of postoperative complications in spinal surgery) of orthopedic nursing require nurses to have strong professional capabilities. However, the role expectation of “expert nurses” from patients and their families easily makes nurses perceive the gap between “ability and expectation,” thereby triggering CFP (11). The core difference between CFP and general occupational stress lies in its “face-threatening” attribute—when dealing with complex cases, orthopedic nurses not only worry about insufficient ability affecting patient rehabilitation but also fear negative evaluations from colleagues and patients. This dual pressure has a significantly higher predictive power for burnout than general stressors (9). Clarifying the relationship between CFP and burnout can provide a basis for nursing managers to develop a two-track intervention strategy of ability recognition and psychological counseling to help reduce the turnover rate of orthopedic nurses. To date, few studies have examined CFP among nurses, particularly those in orthopedic settings. Limited evidence exists for cardiothoracic nurses (12) and ICU nurses (13); these authors suggest that reducing CFP while simultaneously increasing job crafting may relieve burnout. For orthopedic nurses, complex clinical judgments, meticulous nursing procedures, and high family expectations may lead them to form negative self-evaluations of insufficient ability, thereby triggering CFP.

Job crafting refers to proactive behaviors aimed at aligning job demands with personal resources by modifying task boundaries, cognitive appraisals, and relational interactions (14, 15). Specifically, it includes task crafting (e.g., optimizing rehabilitation guidance processes), cognitive crafting (e.g., viewing complex cases as opportunities for ability improvement), and relational crafting (e.g., strengthening collaboration with physicians), which help nurses achieve resource supplementation (16). Empirical studies have shown that job crafting can mitigate burnout among emergency department nurses by enhancing their perceived control and professional value (17, 18). According to the Conservation of Resources Theory, CFP consumes nurses’ psychological resources (such as confidence and enthusiasm) and inhibits their motivation for job crafting; while job crafting can buffer the negative impact of CFP on burnout by supplementing ability resources, psychological resources, and social resources, thus having a mediating role (19). This study regards job crafting as a mediating variable rather than a moderating variable because the core hypothesis is that CFP indirectly affects burnout by influencing job crafting behaviors—rather than job crafting moderating the direct relationship between CFP and burnout—which is consistent with the causal path logic between variables (20).

Existing studies have confirmed the impact of CFP on healthcare workers’ burnout, but three gaps remain. Few studies have focused on orthopedic nurses—orthopedic nursing involves complex clinical judgments (e.g., postoperative complication identification), high-precision rehabilitation operations (e.g., functional training after joint replacement), and high family expectations, which easily widen the gap between ability and expectations, thereby leading to prominent CFP. However, the pressure characteristics of this group have not been systematically explored. The mediating role of job crafting has not been verified in orthopedic settings—job crafting, as a set of behaviors involving proactive adjustment of work tasks, cognition, and interpersonal relationships, has been shown to alleviate nurses’ burnout. But whether it can buffer the impact of CFP on orthopedic nurses’ burnout and its internal mechanism remains unclear; a lack of targeted intervention basis—clarifying the mediating mechanism—can provide nursing managers with a dual intervention approach of “stressor management and resource activation.”

According to the Conservation of Resources Theory (21), the core assumption is that “resource loss takes precedence over resource gain.” An individual’s occupational health depends on the balance between resource acquisition and consumption. If individuals continuously face resource threats (such as psychological resource consumption caused by CFP) in occupational settings, burnout will be triggered, while active resource acquisition (such as ability improvement and social support brought by job crafting) can alleviate this process. CFP is essentially a “resource threat”—when nurses perceive that their abilities cannot meet professional requirements, it leads to the consumption of psychological resources, thereby triggering burnout (direct path). Job crafting can supplement resources through task optimization (acquiring ability resources), cognitive adjustment (activating psychological resources), and relationship building (obtaining social resources), buffering the impact of pressure on burnout (indirect path), which provides a theoretical basis for the hypotheses of this study. Based on the aforementioned evidence, this study aimed to (a) assess the levels and correlates of CFP, job crafting, and burnout among orthopedic nurses; (b) examine the mediating role of job crafting; and (c) inform the development of targeted intervention strategies.

Existing studies have confirmed the correlation between CFP and burnout in ICU nurses and cardiac surgery nurses (12, 13) but have not focused on the high-precision and long-cycle nursing scenarios of orthopedic nurses; the mediating role of job crafting has only been verified in emergency nurses (17) and has not been clarified in orthopedic scenarios. This study is the first to include CFP, job crafting, and burnout in the same framework to verify the relationship and mediating mechanism among the three in orthopedic nurses and to fill the research gap regarding the CFP impact mechanism in specialized nursing scenarios. Hypotheses are as follows: H1: CFP is positively associated with burnout; H2: CFP is negatively associated with job crafting; H3: Job crafting is negatively associated with burnout; H4: Job crafting mediates the relationship between CFP and burnout. The results of this study are of great significance to multiple parties: for nursing managers, it can identify key influencing factors of orthopedic nurses’ burnout and optimize personnel management strategies (e.g., targeted psychological support); for health administrators, it can provide data support for formulating orthopedic nursing human resource protection policies; and for policymakers, it can be incorporated into the nursing occupational health protection system to promote the improvement of specialized nursing quality.

## Materials and methods

### Participants

This study was approved by the Human Research Ethics Committee of Aba Teachers College (Approval No. 202509). A multistage cluster sampling method was adopted. In Stage 1, 10 Grade A tertiary hospitals were randomly selected from Sichuan Province (3 in the provincial capital and 7 in prefecture-level cities). In Stage 2, all nurses meeting the inclusion criteria were recruited from the orthopedic wards of the selected hospitals as cluster units to ensure sample representativeness. The inclusion criteria were as follows: (a) holding a valid registered nurse license; (b) having a minimum of 3 years of clinical experience in orthopedic nursing (including permanent placement following rotation); (c) having no absence from clinical duties in the past month; and (d) being willing to provide written informed consent. All procedures were conducted in accordance with the Declaration of Helsinki. Exclusion criteria included the following: (a) holding a managerial position in orthopedic nursing (e.g., head nurse or administrative role); (b) presence of psychiatric or severe physical conditions that could interfere with participation; and (c) current enrollment in advanced training or residency programs.

Orthopedic nurses with at least 3 years of clinical experience were included because professional proficiency in orthopedic nursing requires more than 3 years of accumulation. This group has independently undertaken core nursing tasks and can accurately perceive CFP (such as pressure in handling complex cases) (22); Rotating nurses and newly recruited nurses were excluded to avoid measurement bias of variables due to unstable work content; according to the mediation sample size calculation formula proposed by Fritz & MacKinnon (20) (considering the number of predictors, mediators, and effect sizes), with an effect size *f*^2^ = 0.15 (medium effect), *α* = 0.05, and statistical power (1 − *β*) = 0.90, a minimum sample size of 110 was required. Considering a 20% invalid questionnaire rate, 240 nurses were recruited, and 216 valid questionnaires were finally collected, meeting the sample size requirement for mediation effect testing.

### Baseline characteristics

Of the 216 nurses, 198 were female (91.67%), and 18 were male (8.33%). Age: < 30 y, *n* = 72 (33.33%); 30–40 y, *n* = 90 (41.67%); ≥ 40 y, *n* = 54 (25.00%). Education: secondary nursing school, *n* = 15 (6.94%); junior college, *n* = 78 (36.11%); bachelor’s degree or above, *n* = 123 (56.94%). Professional title: Junior, *n* = 96 (44.44%); intermediate, *n* = 99 (45.83%); senior, *n* = 21 (9.72%). Years of experience: <5 y, *n* = 81 (37.50%); 5–10 y, *n* = 75 (34.72%); ≥10 y, *n* = 60 (27.78%). Average monthly income: <RMB 10,000, *n* = 84 (38.89%); RMB 10,000–15,000, *n* = 93 (43.06%); ≥RMB 15,000, *n* = 39 (18.06%). Average monthly night shifts: 0–3, *n* = 24 (11.11%); 4–5, *n* = 132 (61.11%); ≥6, *n* = 60 (27.78%). Marital status: unmarried, *n* = 75 (34.72%); married/divorced, *n* = 141 (65.28%). Employment status: permanent, *n* = 51 (23.61%); contract, *n* = 165 (76.39%).

### Measures

Data collection was conducted by trained research assistants: 1. Questionnaire distribution: Anonymous questionnaires were distributed one-on-one during non-peak working hours in orthopedic wards. 2. On-site conditions: After explaining the study purpose and confidentiality commitments, research assistants left the site to allow participants to fill out the questionnaires independently, avoiding social desirability bias caused by the presence of supervisors/colleagues. 3. Item clarification: If participants had questions while filling out the form, research assistants only explained the meaning of the items according to unified guidelines without providing directional hints. 4. Recovery and verification: Questionnaires were collected immediately after completion, and their completeness was checked. Missing items were reminded to be supplemented immediately.

A general information questionnaire was used to collect data on the participants’ sex, age, education level, professional title, years of clinical experience, average monthly income, number of night shifts per month, marital status, and employment type.

The CFP Scale, a four-item Chinese scale adapted for nurses by Chang et al. (23), was used to assess perceived pressure associated with competence and face-saving concerns. A sample item is “I worry that my colleagues look down on me because of my insufficient ability.” Items are rated on a 5-point Likert scale ranging from 1 (strongly disagree) to 5 (strongly agree), with total scores ranging from 4 to 20. Higher scores indicate greater perceived pressure. In this study, the scale demonstrated good internal consistency (Cronbach’s *α* = 0.829; Table 1).

**Table 1:** Reliability coefficients of each scale (*n* = 216).

Scale	Cronbach’s α coefficient	Cronbach’s α coefficients of dimensions
Capability face pressure scale	0.829	—
Job crafting scale	0.832	Task crafting (0.816), cognitive crafting (0.795), relational crafting (0.802)
Maslach burnout inventory	0.830	Emotional exhaustion (0.812), depersonalization (0.788), reduced personal accomplishment (0.796)

All reliability coefficients are >0.7, meeting psychometric requirements.

The Job Crafting Scale, a 21-item Chinese version revised for nurses by Cheng et al. (22), covers three dimensions: task crafting (7 items, e.g., “I proactively add nursing tasks that promote patient rehabilitation”), cognitive crafting (7 items, e.g., “I view complex nursing work as an opportunity to improve my ability”), and relational crafting (7 items, e.g., “I actively communicate with physicians to optimize patient care plans”). Likert 5-point (1 = never, 5 = always); total score 21–105, higher = greater crafting. Cronbach’s *α* was 0.832 overall and 0.795–0.816 for subscales (Table 1).

Maslach Burnout Inventory: 22-item Chinese-nurse version revised by Lee et al. (24) with three dimensions—emotional exhaustion (9 items, e.g., “I feel emotionally drained from my work”), depersonalization (5 items, e.g., “I have become more callous toward patients’ needs”), and reduced personal accomplishment (8 items, e.g., “I feel satisfied with my nursing work” [reverse scored]). Likert 7-point (1 = never, 7 = always); total score 0–132, higher = greater burnout. Cronbach’s *α* was = 0.830 overall and 0.788–0.812 for subscales (Table 1).

### Statistical analysis

Data were analyzed using SPSS version 26.0. Continuous variables with normal distribution are presented as means ± standard deviations. Group comparisons were conducted using independent-samples *t*-tests for two groups and one-way analysis of variance (ANOVA) for multiple groups.

Pearson correlation analysis was used to test the correlation between variables because the data conformed to normal distribution (K-S test *p* > 0.05); Hayes PROCESS macro (Model 4) was used to test the mediating effect to verify the chain path of “independent variable → mediating variable → dependent variable.” The cut-off scores for the scales were defined as follows: a total score of ≥14 on the CFP Scale indicates high pressure (23); the total score of the Job Crafting Scale ≥85 indicates high-level crafting (22); the total score of the Burnout Scale ≥60 indicates high burnout (24). A path model was constructed using AMOS version 23.0, and standardized path coefficients were used to estimate the effect sizes. The fit indices of the structural equation model included χ^2^/df, GFI, AGFI, RMSEA, TLI, CFI, and SRMR. A significance level of *α* = 0.05 was set.

## Results

### Overall status

The total scores for CFP, job crafting, and burnout among orthopedic nurses are shown in Table 2. The mean CFP score among the 216 orthopedic nurses was 13.86 ± 2.62 (item-level mean = 3.46 ± 0.65). The total job crafting score was 88.72 ± 14.95 (item mean = 4.22 ± 0.71). The subscale means were as follows: task crafting = 30.12 ± 5.68, cognitive crafting = 27.86 ± 5.12, and relational crafting = 29.74 ± 5.05. The total burnout score was 58.64 ± 10.52 (item mean = 2.67 ± 0.48). The subscale means were emotional exhaustion = 29.12 ± 5.56, depersonalization = 11.86 ± 3.62, and reduced personal accomplishment = 16.98 ± 4.08.

**Table 2:** Scores of capability face pressure, job crafting, and burnout among orthopedic nurses (*n* = 216).

Variable	No. of items	Total score	Mean item score
Capability face pressure	4	13.86 ± 2.62	3.46 ± 0.65
Job crafting	21	88.72 ± 14.95	4.22 ± 0.71
Task crafting	7	30.12 ± 5.68	4.30 ± 0.81
Cognitive crafting	7	27.86 ± 5.12	3.98 ± 0.73
Relational crafting	7	29.74 ± 5.05	4.25 ± 0.72
Burnout	22	58.64 ± 10.52	2.67 ± 0.48
Emotional exhaustion	9	29.12 ± 5.56	3.24 ± 0.62
Depersonalization	5	11.86 ± 3.62	2.37 ± 0.72
Reduced personal accomplishment	8	16.98 ± 4.08	2.12 ± 0.51

CFP differed significantly across sex, age, education level, professional title, years of experience, monthly income, and number of night shifts per month (all *p* < 0.05). No significant differences were found in relation to marital status or employment type (*p* > 0.05). Male nurses reported higher scores than did female nurses. Nurses aged ≥40 years scored higher than those aged <30 years. Individuals with a bachelor’s degree or higher reported greater pressure than those with secondary education. Senior nurses scored higher than junior nurses. Those with ≥10 years of experience reported more pressure than those with <5 years of experience. Nurses earning ≥RMB 15,000 per month reported higher scores than those earning <RMB 10,000 per month. Finally, nurses working 0–3 night shifts reported higher pressure than those working ≥6 night shifts (Table 3). Unexpectedly, nurses with 0–3 night shifts per month reported significantly higher CFP than those with ≥6 night shifts (*p* < 0.01). Further analysis of confounding variables revealed that nurses with fewer night shifts were mostly senior nurses (75% with ≥10 years of experience), who needed to independently handle night emergencies (e.g., postoperative massive hemorrhage) and take on additional responsibilities such as departmental teaching and emergency guidance, leading to higher expectations for their ability. Nurses with more night shifts were mainly junior nurses (82% with junior professional titles), whose work focused on routine nursing with sufficient team support, resulting in lower CFP. This result suggests that the impact of night shift frequency on pressure should be comprehensively considered in combination with position and work content, rather than simply “more night shifts = higher pressure.”

**Table 3:** Comparison of capability face pressure, job crafting, and burnout by demographic and occupational characteristics.

Parameter	Group	*n*, %	Capability face pressure score	t/F	*p*	Job crafting score	t/F	*p*	Burnout score	t/F	*p*
Gender	Male	18 (8.33)	16.24 ± 3.15	3.286	<0.01	90.15 ± 15.02	0.452	>0.05	56.82 ± 10.24	0.985	>0.05
	Female	198 (91.67)	13.62 ± 2.54			88.56 ± 14.88			58.84 ± 10.56		
Age (years)	<30	72 (33.33)	12.15 ± 2.48	11.682	<0.01	84.26 ± 14.52	8.954	<0.01	57.28 ± 10.32	4.256	<0.01
	30 to <40	90 (41.67)	13.92 ± 2.65			88.95 ± 15.01			60.84 ± 10.68		
	≥40	54 (25.00)	15.86 ± 2.98			94.12 ± 15.68			55.62 ± 10.15		
Education	Secondary	15 (6.94)	12.08 ± 2.56	10.953	<0.01	87.92 ± 14.76	0.218	>0.05	58.15 ± 10.42	0.196	>0.05
	Junior College	78 (36.11)	13.05 ± 2.62			88.36 ± 14.92			58.92 ± 10.58		
	Bachelor’s or above	123 (56.94)	14.86 ± 2.85			89.15 ± 15.06			58.42 ± 10.48		
Professional title	Junior	96 (44.44)	12.85 ± 2.58	9.862	<0.01	83.65 ± 14.62	7.528	<0.01	57.12 ± 10.38	5.124	<0.01
	Intermediate	99 (45.83)	13.96 ± 2.72			88.72 ± 14.98			61.25 ± 10.72		
	Senior	21 (9.72)	15.68 ± 2.95			95.24 ± 15.85			54.86 ± 10.05		
Years of experience	<5	81 (37.50)	12.78 ± 2.56	12.158	<0.01	84.15 ± 14.58	9.256	<0.01	56.85 ± 10.28	4.862	<0.01
	5 to <10	75 (34.72)	13.86 ± 2.68			88.52 ± 14.95			60.98 ± 10.65		
	≥10	60 (27.78)	15.52 ± 2.92			93.86 ± 15.72			55.24 ± 10.12		
Monthly income (RMB)	<10,000	84 (38.89)	12.05 ± 2.48	18.956	<0.01	85.24 ± 14.65	6.852	<0.01	56.92 ± 10.32	5.586	<0.01
	10,000 to <15,000	93 (43.06)	13.92 ± 2.65			88.65 ± 14.98			61.58 ± 10.75		
	≥15,000	39 (18.06)	15.98 ± 2.96			94.52 ± 15.88			54.65 ± 10.08		
Night shifts per month	0–3	24 (11.11)	15.72 ± 2.95	8.562	<0.01	94.86 ± 15.92	7.125	<0.01	54.25 ± 10.06	5.248	<0.01
	4–5	132 (61.11)	13.65 ± 2.62			88.25 ± 14.92			58.96 ± 10.52		
	≥6	60 (27.78)	12.85 ± 2.58			84.52 ± 14.56			61.85 ± 10.82		
Marital status	Unmarried	75 (34.72)	14.02 ± 2.68	0.528	>0.05	85.12 ± 14.65	2.456	<0.05	57.86 ± 10.42	1.358	>0.05
	Married/divorced	141 (65.28)	13.75 ± 2.61			89.85 ± 15.02			59.02 ± 10.58		
Employment type	Permanent	51 (23.61)	13.68 ± 2.58	0.685	>0.05	88.92 ± 14.95	0.072	>0.05	58.12 ± 10.38	0.625	>0.05
	Contract	165 (76.39)	13.92 ± 2.65			88.65 ± 14.98			58.85 ± 10.56		

Job crafting differed significantly by age, professional title, years of experience, average monthly income, average monthly number of night shifts, and marital status (*p* < 0.05). Sex, education, and employment type showed no significant differences (*p* > 0.05). Nurses ≥40 years scored higher than those <30 years; senior titles scored higher than junior titles; those with ≥10 years of experience scored higher than those with <5 years of experience; income ≥RMB 15,000 scored higher than those with an income <RMB 10,000; those with 0–3 night shifts scored higher than those with ≥6 night shifts; and those who were married/divorced scored higher than those who were unmarried (Table 3).

Burnout differed significantly by professional title, years of experience, average monthly income, and average monthly number of night shifts (*p* < 0.05). Sex, education, marital status, and employment type showed no significant differences (*p* > 0.05). Nurses aged 30–40 years scored higher than those aged <30 years and ≥40 y; intermediate-title nurses scored higher than junior and senior nurses; nurses aged 5–10 years scored higher than those aged <5 years and ≥10 years; nurses aged 10,000–15,000 scored higher than those aged <RMB 10,000 and ≥RMB 15,000, and nurses aged ≥ 6 night shifts scored higher than those aged 0–3 night shifts (Table 3).

### Correlation analysis

As shown in Table 4, CFP was significantly negatively correlated with all dimensions of job crafting (*r* = −0.528 to −0.603, *p* < 0.01), indicating that the higher the capability face pressure of orthopedic nurses, the lower their willingness to actively adjust work tasks, cognition, and interpersonal relationships. Burnout was significantly positively correlated with CFP and negatively correlated with job crafting, suggesting that both variables may play important roles in predicting nurses’ burnout.

**Table 4:** Correlation analysis of capability face pressure, job crafting, and burnout among orthopedic nurses (*n* = 216).

Variable	1	2	3	4	5	6	7	8	9
1. Capability face pressure	1.000								
2. Job crafting	−0.603**	1.000							
3. Task crafting	−0.578**	0.825**	1.000						
4. Cognitive crafting	−0.562**	0.788**	0.528**	1.000					
5. Relational crafting	−0.528**	0.802**	0.605**	0.702**	1.000				
6. Burnout	0.658**	−0.576**	−0.472**	−0.485**	−0.465**	1.000			
7. Emotional exhaustion	0.605**	−0.538**	−0.408**	−0.442**	−0.415**	0.808**	1.000		
8. Depersonalization	0.583**	−0.502**	−0.421**	−0.388**	−0.392**	0.725**	0.578**	1.000	
9. Reduced personal accomplishment	−0.412**	0.458**	0.486**	0.425**	0.402**	−0.508**	−0.325**	−0.398**	1.000

**p < 0.01.

With CFP as the independent variable (*X*), burnout as the dependent variable (*Y*), and job crafting as the mediator (M), after controlling for sex, age, professional title, etc., regression analysis showed that the direct effect of CFP on burnout was significant (*β* = 0.565, SE = 0.092, *t* = 6.141, *p* < 0.001). Effect of CFP on job crafting (*β* = −0.603, SE = 0.085, *t* = −7.094, *p* < 0.001). Combined effects: CFP (*β* = 0.322, SE = 0.025, *t* = 12.880, *p* < 0.001) and job crafting (*β* = −0.398, SE = 0.068, *t* = −5.853, *p* < 0.001) were both significantly associated with burnout.

The bootstrap mediation test results are presented in Table 5. The total effect was 0.567 (95% CI: 0.382–0.752), the direct effect was 0.324 (95% CI: 0.275–0.373), and the indirect effect was 0.243 (95% CI: 0.052–0.434), accounting for 42.87% of the total effect. All 95% CIs excluded zero, indicating significant partial mediation.

**Table 5:** Mediating effect of job crafting between capability face pressure and burnout.

Effect type	*β*	SE	95% CI	Proportion of total effect (%)
Total effect	0.567	0.093	0.382–0.752	—
Direct effect	0.324	0.025	0.275–0.373	57.13
Indirect effect	0.243	0.098	0.052–0.434	42.87

**Figure 1:**
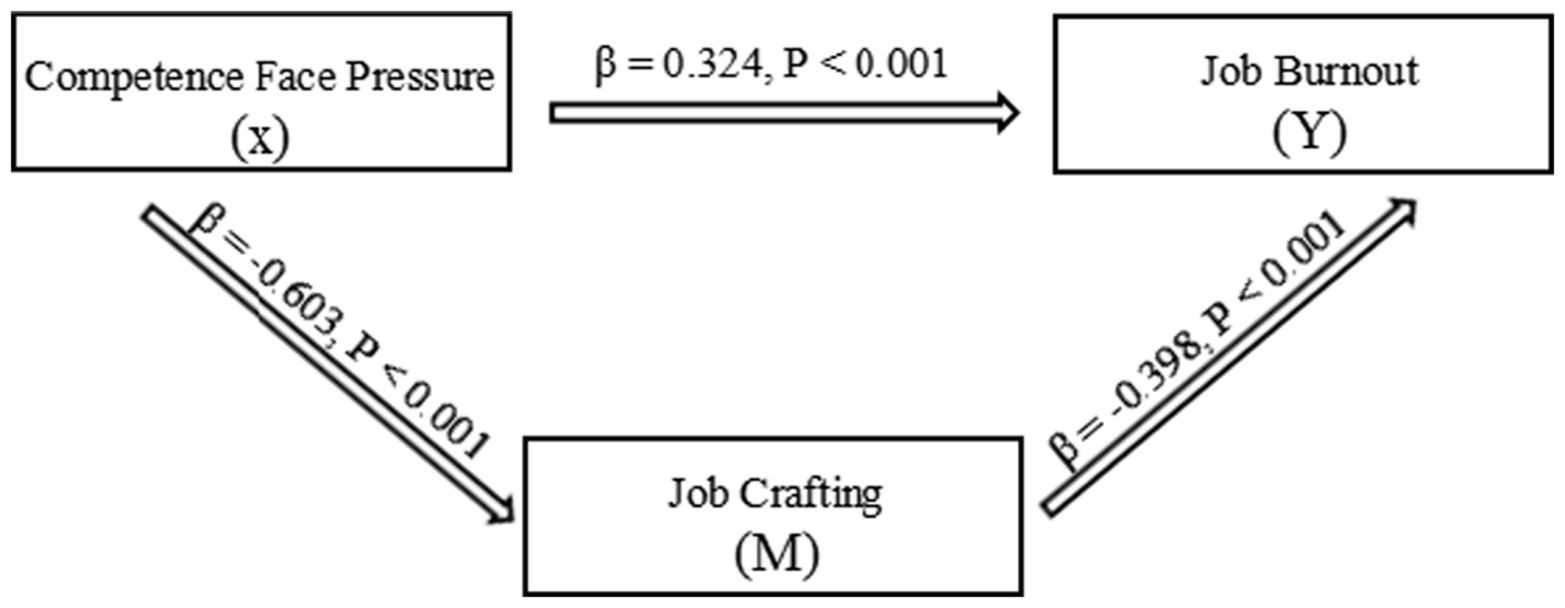
Path diagram of the mediating role of job crafting between capability face pressure and burnout.

The fit results of the structural equation model were χ^2^/df = 1.852, GFI = 0.925, AGFI = 0.898, RMSEA = 0.062, TLI = 0.931, CFI = 0.945, and SRMR = 0.058. Among them, TLI > 0.9, CFI > 0.9, SRMR < 0.08, and RMSEA < 0.08, indicating a good model fit. The path model (Figure 1) demonstrated a good fit. Standardized coefficients: CFP was significantly associated with burnout (*β* = 0.324, *p* < 0.001); CFP was significantly associated with job crafting (*β* = −0.603, *p* < 0.001); job crafting was significantly associated with burnout (*β* = −0.398, *p* < 0.001).

## Discussion

This study aims to explore the relationship between CFP, job crafting, and burnout in orthopedic nurses and verify the mediating role of job crafting. The core findings show that CFP is significantly positively correlated with burnout (*r* = 0.658); CFP is significantly negatively correlated with job crafting (*r* = −0.603); job crafting is significantly negatively correlated with burnout (*r* = −0.576); job crafting partially mediates the relationship between CFP and burnout, with the indirect effect accounting for 42.87% of the total effect; and nurses aged 30–40, with intermediate professional titles, and frequent night shifts have the highest burnout level.

First, CFP was significantly positively associated with burnout, consistent with findings among ICU nurses (13) and cardiothoracic nurses (12). This finding suggests that CFP is an important factor associated with burnout across various high-risk nursing specialties. Orthopedic nurses often face complex clinical decisions, such as identifying postoperative complications, and meet demanding rehabilitation needs. When they doubt their competence, such pressures may lead to anxiety, emotional exhaustion, and eventual depersonalization (3, 4). Nurses aged 30–40 have the highest burnout because this group undertakes clinical nursing, teaching, and family responsibilities simultaneously, with overlapping multiple pressures (13). Nurses with fewer night shifts have higher CFP because most of them are senior nurses who need to independently handle night emergencies with higher ability expectations (11). Intermediate-title nurses have higher burnout, which may be related to the “mid-career crisis”—they are expected to have professional proficiency but often lack sufficient resources and support compared to senior nurses. Second, CFP was significantly negatively associated with job crafting, a finding consistent with the Conservation of Resources theory (19, 25). High levels of CFP may deplete psychological resources such as confidence and enthusiasm, undermining nurses’ motivation to proactively adjust their work tasks, cognitive appraisals, or relational interactions—key components of job crafting. Third, job crafting was significantly negatively associated with burnout, a finding that corroborates previous research among health center nurses (26) and male nurses (27). Task crafting (e.g., optimizing nursing procedures), cognitive crafting (e.g., viewing challenges positively), and relational crafting (e.g., enhancing physician collaboration) increase perceived control and professional value, alleviating burnout. Finally, job crafting significantly mediated the relationship between CFP and burnout (indirect effect = 0.243, 42.87% of the total effect). These findings suggest that CFP is not only directly associated with burnout but also indirectly undermines well-being by reducing job crafting behaviors, highlighting a potential target for intervention.

With respect to demographic differences, male nurses and those with higher education or senior professional titles reported significantly greater CFP. These findings are consistent with those reported by Gong et al. (11) among male general practitioners. Male nurses, as a minority in a female-dominated field, may face heightened face threat when demonstrating competence. Similarly, nurses with higher academic qualifications or senior titles often encounter greater external expectations, such as teaching and research responsibilities, that widen the gap between perceived ability and role demands. All job crafting dimensions were negatively associated with burnout, confirming the universal protective effect reported previously (26, 28).

Notably, nurses aged 30–40 years reported the highest levels of burnout, a finding that contradicts a meta-analysis, which reported no significant age-related differences (29). This age group often serves as the backbone of clinical units, balancing direct patient care, mentorship roles, and family responsibilities, factors that may compound occupational stress. In contrast, cardiothoracic nurses with ≥40 years of experience have high burnout due to intense intraoperative cooperation and emergency tasks. Surprisingly, nurses working 0–3 night shifts per month reported higher CFP than those working ≥6 shifts, challenging the common assumption that more night shifts inevitably lead to greater stress. One possible explanation is that nurses with fewer night shifts are often senior staff who must independently manage critical events—such as postoperative hemorrhage—during infrequent night duties, thereby experiencing greater face threat. In contrast, those working more night shifts were typically junior nurses performing routine tasks with stronger team support, resulting in lower perceived pressure.

Direct Impact Mechanism of CFP on Burnout: The strong positive correlation between CFP and burnout among orthopedic nurses was mainly due to the high-risk and high-precision characteristics of orthopedic nursing (e.g., postoperative care for scoliosis correction and deep vein thrombosis prevention). Nurses face pressure that an insufficient ability may lead to serious consequences. When they perceived that their ability could not meet these requirements, they experienced continuous anxiety (psychological resource depletion), leading to emotional exhaustion; to avoid exposing their incompetence, nurses might adopt an emotionally distant attitude towards patients (reducing active communication), reinforcing depersonalization; long-term negative self-evaluation reduced professional accomplishment, ultimately forming burnout syndrome.

The underlying mechanism can be conceptualized as a dual-pathway model. In the direct pathway, CFP arises from a perceived mismatch between one’s actual abilities and external face-saving demands (30). When nurses perceive themselves as unable to meet role expectations, such as providing precise rehabilitation guidance following joint replacement, they experience face threat. This perceived inadequacy constantly depletes emotional resources, leading to emotional exhaustion. To avoid revealing their limitations, nurses may adopt emotionally distant attitudes, further reinforcing depersonalization. In the indirect pathway, a high CFP inhibits job crafting via resource depletion (31). Fear of incompetence discourages them from attempting new nursing tasks (reduced task crafting), fosters negative appraisals of challenges (reduced cognitive crafting), and limits their interaction with colleagues or patients because of fear of communication errors (reduced relational crafting). Declining job crafting further weakens resource acquisition (16). Conversely, task crafting enhances capability resources (32), cognitive crafting optimizes psychological resources (33), and relational crafting secures social resources (34), jointly offsetting the negative impact of CFP and alleviating burnout.

The result that nurses with fewer night shifts had higher CFP was inconsistent with previous studies concluding that night shift frequency is positively correlated with CFP, but it can be explained by clinical practice and literature support. On the one hand, senior nurses (who mostly take fewer night shifts) are required to independently handle night emergencies and face social expectations that senior staff should have a higher ability. This role expectation pressure has been proven to increase healthcare workers’ face pressure (11); on the other hand, sensitivity analysis (re-testing after excluding nurses with ≤5 years of work experience) showed that the result was still significant, proving that it was not caused by sample composition bias and was stable.

This study has the following limitations: A cross-sectional design was used, which cannot establish causal relationships between variables but only reveal correlations; samples were from 10 hospitals in Sichuan Province, with a single region, which may affect the external validity of the results; self-report scales were used, which may lead to social desirability bias; potential moderating variables such as psychological capital and social support were not included, and the model integrity needs to be improved. Future research can adopt a longitudinal follow-up design (1-year follow-up) to verify the causal path of CFP to job crafting and burnout; expand the sample scope to multiple regions nationwide, including hospitals of different levels, to improve the representativeness of results; introduce psychological capital as a moderating variable to construct an expanded model of CFP-job crafting-psychological capital-burnout; and develop targeted intervention programs (job crafting training and CFP psychological counseling) and verify their effects.

## Conclusion

This study is the first to systematically investigate the relationship between CFP, job crafting, and burnout among orthopedic nurses. This confirms that CFP is an important factor associated with burnout, and job crafting plays a partial mediating role (accounting for 42.87% of the total effect). Burnout prevention efforts should focus on reducing CFP through psychological support and competency recognition and validation, and enhancing job crafting through targeted training and collaborative support. Special attention is warranted for nurses aged 30–40 years, those with intermediate professional titles, and those working infrequently on night shifts. The contributions of this study are reflected in three aspects: It extends the construct of CFP to the field of orthopedic nursing and verifies the applicability of the Conservation of Resources Theory in specialized nursing groups; it proposes a dual intervention framework of stressor control and resource activation to provide operable strategies for nursing managers; and it provides data support for formulating orthopedic nursing human health protection policies and helps continuously improve specialized nursing quality.

## Data Availability

The raw data supporting the conclusions of this article will be made available by the authors, without undue reservation.
